# Frailty in elderly patients with acute appendicitis

**DOI:** 10.1007/s00068-022-01878-2

**Published:** 2022-02-02

**Authors:** Alexander Reinisch, Martin Reichert, Christian Charles Ondo Meva, Winfried Padberg, Frank Ulrich, Juliane Liese

**Affiliations:** 1 Department of General, Visceral and Oncologic Surgery, Hospital and Clinics Wetzlar; Teaching Hospital of the JLU Giessen, Wetzlar, Germany; 2 Department of General, Visceral, Thoracic, Transplant and Pediatric Surgery, University Hospital Giessen, Giessen, Germany; 3 Department of General, Visceral and Oncologic Surgery, Dill-Clinics, Dillenburg, Germany

**Keywords:** Appendicitis, Frailty, Surgery, Geriatric

## Abstract

**Purpose:**

Acute appendicitis in the elderly is becoming increasingly recognized for its often severe course. For various elective and urgent operations in older patients, frailty is a risk factor for poor outcomes. However, there is a lack of data on frailty in elderly patients with acute appendicitis.

**Methods:**

Patients over 65 years old who underwent surgery for acute appendicitis in three hospitals between January 2015 and September 2020 were assessed with the Hospital Frailty Risk Score (HFRS) and the modified Frailty Index (mFI). Outcomes of interest, including morbidity, mortality, and length of stay, were recorded.

**Results:**

While frailty can be measured with both tests, the mFI has better applicability and takes significantly less time to implement compared to the HFRS (21.6 s vs. 80.3 s, *p *< 0.0001) while providing the same information value.

Patients who exhibited frailty according to either assessment had a significantly higher rate of milder (OR 5.85/2.87, *p *< 0.0001/0.009) and serious (OR 4.92/3.61, *p *< 0.011/0.029) complications, more admissions to the intensive care unit (OR 5.16/7.36, *p *< 0.0001), and an almost doubled length of stay (12.7 days vs. 6.6 days, *p *< 0.005). Up to 31% of these patients required institutional care after discharge, which is significantly more than those without frailty (*p *< 0.0001). Furthermore, the mortality rate in frail patients was significantly elevated to 17%, compared to less than 1% in non-frail patients (*p *= 0.018).

**Conclusion:**

In elderly patients, frailty is a significant risk factor for negative outcomes. Frailty can be assessed more quickly and reliably with the mFI compared to the HFRS.

## Introduction

The unique challenges of treating elderly patients with acute appendicitis are not obvious. However, it is becoming increasingly understood that acute appendicitis, despite its maximum incidence in young adulthood, is a significant illness with very unfavorable outcomes in the elderly population [1, 2]. As the number of elderly individuals, mostly defined as people over 65 years of age, is continuously increasing in developed countries, acute appendicitis in the elderly and its associated challenges will become more prevalent [3, 4]. Thus, the long-prevailing notion that appendicitis is a disease of the young is partly outdated [5].

Acute appendicitis in the elderly differs from that in young people; more complicated appendicitis with perforations is observed in older patients [6]. The more decisive factor, however, is the association between old age and poor outcomes. Among other challenges, older patients suffer from a complication rate of up to 25%, require longer hospital treatment than younger patients, and have a marked mortality rate of up to 8–16% [4, 7, 8].

This illustrates the particular importance of understanding appendicitis in older people, a fact that is also reflected in issuing a special guideline for appendicitis in older people [9].

Special and unfavorable courses in the treatment of older people are explained less by the *numerical age* than by the *biological age*. For some elderly patients, the risk of an unfavorable outcome of treatment goes far beyond the risk inherent in the disease and higher age itself [10]. An approximation to *biological age* can be achieved in part by measuring *frailty*. Frailty has established itself as a multidimensional syndrome and can be summarized as an age-related “consequence of dysregulation in several physiological control circles”, leading to lower resilience and increased vulnerability to stressors [11, 12]. The *physical phenotype model of frailty* (Fried) includes physical aspects and mental and social factors but the scope and weighting of these factors are not clearly defined.

Emergency surgery is a significant stressor even for young and healthy patients. Frail patients are even more vulnerable and less resilient to emergency surgery as it has been shown that frailty is an independent risk factor for negative outcomes. This applies not only for medical factors (e.g., complications) but also for social aspects, such as the opportunity to return to one’s usual living environment [13]. Few studies have investigated the role of frailty in emergency general surgery patients. In a recent study, Fagenson et al. showed that frailty is a factor for negative outcomes in acute cholecystitis. Murphy et al. and Sánchez Arteaga et al. evaluated different emergency general surgery operations and found similar results [14, 15]. Other studies have shown that frailty is a risk factor for, inter alia, morbidity and mortality [16–19]. However, either unreported or playing only a subordinate role in these studies, appendicitis has yet to be fully addressed.

Frailty can be framed as “reduced performance” (Fried) and as an “accumulation of deficits” (Rockwood) [12, 20]. There are over 70 validated assessments available to determine frailty, however, none of which have been established as the gold standard. Approximately half of these have been used on surgical patients [21]. Many established tests are not suitable for patients in an acute situation because they often refer to a period of several weeks before testing and are biased by acute illness. Several other tests (e.g., Edmonton Frail Scale, CSHA-Frailty index, LUCAS) require the active participation of the patient and are often complex and time-consuming [20, 22, 23]. Easily scaled assessments (e.g., the CSHA scale) are relatively subjective and more suitable for situations in which the patient can be assessed by a family doctor over a long period.

To investigate possible relationships between frailty and acute appendicitis, we had to choose assessments that were retrospectively feasible with routinely recorded data. After the pool of possible assessments was narrowed due to the above restrictions, we chose two tools for this study: (1) the *Hospital Frailty Risk Score (HFRS)* and (2) the *modified Frailty Index (mFI)*, where the latter is relatively simpler than the former.

The mFI [20, 24] records 11 parameters (mFI-11) in the classic version, but a simplified form with only 5 parameters is available (mFI-5) [25]. The advantage of the mFI is that it is not biased by the patient's acute illness and can be recorded by non-geriatric specialists based on information available in the emergency room. This also applies to the HFRS. While the HFRS has the same requirements as the mFI, it is much more complex with over 100 parameters of different weight [26].

The aim of this study was to analyze frailty as a possible risk factor in the treatment of elderly patients with acute appendicitis. Identifying high-risk patients during admission with a reliable assessment can, if necessary, optimize the peri- and postoperative care of these patients.

## Methods

We conducted a retrospective analysis of all patients who underwent surgery for acute appendicitis at one of three participating centers between January 1, 2015, and September 30, 2020. All data were extracted from electronic patient records. Frailty assessment was conducted based on emergency room recording sheets, premedication sheets, and findings made at admission. Only information that was available to the admitting doctor in the emergency room was used in the frailty assessment.

### Inclusion criteria

- Admission and operation for acute appendicitis.
- Age of 65 years or older on the day of admission.
- Complete hospital record including laboratory results, pathological results, insurance data, and data on abidance after dismission.
- Possibility of a complete follow-up of 90 days after the operation.
- Readmitted patients: cause of readmission (surgical vs. alternative).

### Exclusion criteria

- Further planned surgery or intervention during follow-up.
- Scheduled inpatient readmission during follow-up.

All patients were assessed independently by two authors (AR and JL). Patients with a discrepant score/index were reassessed by another randomly selected author, and the majority vote (two out of three) was used. All patients could be evaluated using this technique.

The time necessary to conduct the HFRS and the mFI was recorded. To minimize errors due to learning effects when performing the assessments, the authors were randomly assigned the patient and the respective test (HFRS or mFI) using a computer algorithm.

The HFRS is based on the *International Classification of Diseases* (ICD) codes. The queried electronic patient files do not often explicitly state the corresponding ICD code; however, the ICD code can be determined using the ICD-10 manual, 10th Revision, German Modification (ICD-10-GM). This back-coding was not taken into account when determining the assessment time.

The text in the electronic patient records and the ICD codes often do not exactly match the codes given in the HFRS, even if the conditions are clearly the same [e.g., M15(0.9)—HFRS 0.4 points vs. M19(0.9)—HFRS 1.5 points]. In addition, very broad diagnoses can be found in the HFRS (e.g., Z91: Personal history of risk factors, not elsewhere classified, R69 Unknown and unspecified causes of morbidity, Z87: Personal history of other diseases and conditions). Comparable inaccuracies can theoretically arise when transferring the electronic patient records to the mFI.

To document these inaccuracies, the accuracy of the diagnosis–assessment–transfer was estimated and classified by the authors according to the following categories: *high* for > 75% exact code agreement, low interpretation of the diagnosis codes; *intermediate* for > 50%—< 75% exact code agreement, significant need for interpretation; and *low* for predominantly interpreted diagnosis codes. Discrepant assessments were reconciled based on a majority decision with the addition of a third reviewer in the abovementioned manner.

The outcomes of interest were mortality; complications according to the Dindo/Clavien classification (CD) of surgical complications [27]; admittance to an intensive care unit (ICU); length of stay in the ICU; overall length of hospital stay (LOS); whether the patient was discharged home, to the same type of facility from which she or he originated or a facility of a higher care level; readmittance within 30 or 90 days after the operation; and readmittance due to a surgical/operation-related or an alternative reason.

The abovementioned outcome variables were analyzed against the following covariates: sex; complicated (perforated/gangrenous) vs. uncomplicated appendicitis (histopathology); open vs. laparoscopic vs. conversion laparoscopic-open surgery; unsuspected histopathological or intraoperative findings; health insurance (private vs. statutory); white blood cell count; and C-reactive protein (CRP) at admission.

Comorbidities were analyzed using the *Charlson Comorbidity Index* (CCI). Since this study focused on patients over the age of 65, the age-adjusted CCI was waived as applying it would introduce bias [28].

### Statistical analysis

The patient demographics were expressed as the mean ± standard deviation (SD) or as the median and range, as appropriate. Fisher’s exact test was used to analyze the differences in categorical variables. The Student’s *t* test was applied to variables that were normally distributed. The Mann–Whitney *U* test was applied to non-normally distributed variables. Binary logistic respective linear regression analysis was conducted to determine odds ratios (OR), and qualitative classification was carried out by *receiver operating characteristic* (ROC) analysis. Unless otherwise indicated, all tests were two tailed, and *p* values < 0.05 were considered significant. All data were analyzed with SPSS, version 27 (IBM, Armonk, NY, USA).

## Results

### Demographic parameters

A total of 2,089 patients were included in the retrospective analysis at three centers. The mean age was 32.7 years (SD 20.2 years, range 2.1–98.6 years). Of these, 213 (10.2%) were 65 years or older at the time of the operation, 32 patients were excluded (see methods section). Between the three centers, there were no significant differences in patient characteristics, operative aspects, or any of the outcome parameters.

The characteristics of the 181 included patients are shown in Table 1.

**Table 1:** Patient characteristics

	*n*	%	Mean (SD; min/max)
Total patients	2089		
included patients ≥ 65 years (“elderly patients”)	181	8.7	
Elderly patients			
Age			
Years			75.8 (7.5; 65.1/97.4)
Sex			
Females	90	49.7	
Males	91	50.3	
Operation			
Laparoscopic	163	90.1	
Conversion	8	4.4	
Open	10	5.5	
Complicated appendicitis^†^	113	60.4	
Unsuspected intraoperative findings^††^			
Any	28	15.5	
Malignancy	14	7.7	
Length of stay			
Days			8.2 (7.7; 0/76)
Morbidity			
Any	34	18.8	
Mortality			
90 d	5	2.8	

*d* days, *min* minimum, *max* maximum, *SD* standard deviation

^†^Perforated or gangrenous appendicitis

^††^In addition to an appendicitis

### Frailty assessment

All included patients were evaluated with the abovementioned frailty assessments. The same scores were assessed by the authors for HFRS in 95.6% of the cases and for mFI-11 or mFI-5 in 97.2% of the cases. A majority decision was obtained for all the remaining cases. Of the 181 patients, 33 (18.2%) exhibited no indication of frailty. The mean score for the HFRS was 3.3 (SD 3.5, range 0–15.9). The mean score for the mFI-11 was 1.7 (SD 1.6, range 0–7) and that for the mFI-5 was 1.2 (SD 1.1, range 0–5). The CCI, which was used as a covariate, had a mean value of 1.7 (SD 2.1, range 0–12). The accuracy of the transferability of the diagnoses from the electronic patient record to the assessments differed significantly. The transferability to mFI-11 hat a “high” accuracy in 96.7% and “intermediate” accuracy in 3.3% of the patients. Meanwhile, the HFRS had a “high” transferability-accuracy in 17.1%, an “intermediate” accuracy in 25.4% and a “low” accuracy in 57.5% of the patients (*p *< 0.0001). The time required in seconds (sec) to carry out the assessment also differed significantly; the HRFS took an average of 80.3 s (SD 53.3 s, range 0–220 s) while the mFI-11 took an average of 21.6 s (SD 10, range 0–45 s, *p *< 0.0001).

Cutoffs were as follows:

- HFRS: ≥ 5—intermediate frailty risk; ≥ 15—high frailty risk.
- mFI-11: 0—no frailty; 1—prefrailty; ≥ 3 frailty.
- mFI-5: 0—no frailty; 1—prefrailty; ≥ 2 frailty.

### Relationship between frailty and the postoperative outcome

For the frailty assessments used, significant correlations between the most important outcome variables after appendectomy and frailty were found.

An HFRS ≥ 5 correlates with significantly more frequent overall and serious complications (CD ≥ I: % vs. 16.8%; *p *< 0.0001 resp. CD ≥ III: 18% vs. 3.8%; *p *= 0.003). Patients with an HFRS ≥ 5 were significantly more likely to be admitted to the ICU postoperatively (56% vs. 15.3%, *p *< 0.0001), and stay longer in the ICU; however, this difference was not significant [mean 7.1 days (SD 14.2 days) vs. 2.3 days (SD 1.8 days), *p *= 0.086]. Ten percent of the patients with HFRS ≥ 5 died within 90 days after the operation (vs. 0% HFRS < 5, *p *< 0.0001). With an HFRS ≥ 5, the LOS was significantly longer [12.7 days (SD 11.1 days) vs. 6.6 days (SD 4.9 days), *p *= 0.001], and these patients were more likely to be discharged to an institution with a higher care level than before admission (31.1% vs. 6.1%, *p *< 0.0001). The readmission rate was not significantly elevated. Only two patients had an HFRS ≥ 15, and both had an unfavorable outcome.

In a subgroup analysis of patients older than 75 years (*n* = 89), the abovementioned differences were confirmed but with slightly different significance values.

Patients with an mFI-11 ≥ 3 were more likely to suffer from complications (CD ≥ I 48.8% vs. 22.9%; *p *= 0.003 resp. CD ≥ III 17.1% vs. 5%, *p *= 0.018) or need admission to the ICU and with a longer stay [58.5% vs. 10.7%; *p *= 0.018, ICU-LOS 4.6 days (SD 12.2 days) vs. 0.4 days (SD 1.2 days), *p *< 0.0001]. Differences in length of ICU stay were found between patients with 1 point vs. 0 points [ICU admittance 33.1% vs. 8.5%, *p *< 0.0001; ICU-LOS 5.5 days (SD 11.5 days) vs. 1.5 days (SD 1 day), *p *= 0.034]. Significantly increased mortality was observed in patients with an mFI-11 ≥ 3 (17% vs. 0.7%, *p *= 0.018). Patients with an mFI-11 ≥ 3 stayed significantly longer in the hospital [12.7 days (SD 12.4 days) vs. 6.9 days (SD 5.1 days), *p *= 0.005] and were more often discharged to a facility with a higher care level (29.7% vs. 7.9%, *p *= 0.001; mFI ≥ 1: 16.3% vs. 2.1%, *p *= 0.001). Significant differences in readmission rates could not be observed using the mFI-11.

An mFI-5 ≥ 2 points was correlated with a greater proportion of patients with complications; the difference was significant for CD ≥ I (41.7% vs. 22.3%, *p *= 0.009) and with a trend for CD ≥ III (CD ≥ III 13.3% vs. 4.9%, *p *= 0.073 n. s.). Patients with an mFI-5 ≥ 2 were significantly more often admitted to the ICU (49.2% vs. 15.7, *p *< 0.0001); however, the length of stay in the ICU did not differ significantly. Mortality was significantly elevated in patients with an mFI-5 ≥ 2 (6.7% vs. 0.8%, *p *= 0.042). The LOS was significantly longer in the mFI-5 ≥ 2 group [11.4 days (SD 11.4 days) vs. 6.7 days (SD 4.2 days), *p *< 0.0001], and these patients were more often discharged to an institution with a higher level of care than before admission (25% vs. 6.7%, *p *= 0.001). Overall, readmissions or readmissions for nonsurgical reasons were not significantly elevated in this subgroup, but there were more readmissions for surgical reasons, most frequently for late-onset postoperative complications (26.7% vs. 14.5%, *p *= 0.043).

The observed effects were subject to univariate and multivariate regression analyses. The covariates tested were age, CCI, complicated vs. uncomplicated appendicitis, operative technique, white blood cell count and/or CRP at admission, and insurance status. No regression analysis was possible for the outcome parameter mortality due to the low number of deceased patients (*n *= 5). The results of the univariate and multivariate regression analysis for outcome variables related to frailty assessments are shown in Table 2. There was no association between the patient´s type of insurance for any of the tests, nor was insurance correlated with differences in the examined outcome parameters.

**Table 2:** Univariate and multivariate regression analysis for outcome variables related to frailty assessments

	Univariate			Multivariate		
	OR	95% CI	*p* value	OR	95% CI	*p* value
Complications CD ≥ 1						
HFRS ≥ 5	7.43	3.59–15.39	< .0001	5.85	2.68–12.77	< .0001
mFI-11 ≥ 1	1.99	.89–4.49	n.s	–	–	–
mFI-11 ≥ 3	3.21	1.55–6.66	.002	2.87	1.3–6.32	.009
mFI-5 ≥ 2	2.49	1.27–4.85	.008	2.36	1.13–4.92	.022
Complications CD ≥ 3						
HFRS ≥ 5	5.53	1.75–17.45	.004	4.92	1.45–16.66	.011
mFI-11 ≥ 1	2.21	.48–10.28	n.s	–	–	–
mFI-11 ≥ 3	3.91	1.29–11.9	.016	3.61	1.14–11.45	.029
mFI-5 ≥ 2	2.95	.974–8.93	n.s	–	–	–
ICU admission						
HFRS ≥ 5	7	3.36–14.58	< .0001	5.16	2.31–11.54	< .0001
mFI-11 ≥ 1	5.31	1.79–15.75	.003	5.44	1.63–18.13	.006
mFI-11 ≥ 3	6.76	3.16–14.48	< .0001	7.36	3.09–17.56	< .0001
mFI-5 ≥ 2	5.19	2.55—10.52	< .0001	6.17	2.69–14.14	< .0001
Discharge to higher care level						
HFRS ≥ 5	6.94	2.6–18.02	< .0001	4.53	1.54–13.27	.006
mFI-11 ≥ 1	8.94	1.17–68.44	.035	5.12	.58–44.99	n.s
mFI-11 ≥ 3	4.92	1.93–12.55	.001	4.46	1.5–13.21	.007
mFI-5 ≥ 2	4.67	1.83–11.93	.001	4.56	1.56–13.28	.006
Prolonged LOS						
HFRS ≥ 5	8.92	3.23–24.12	< .0001	4.39	1.36–14.22	.014
mFI-11 ≥ 1	1.56	.49–4.9	n.s	–	–	–
mFI-11 ≥ 3	6.02	6.02–2.32	< .0001	6.01	1.46–24.74	.013
mFI-5 ≥ 2	3.91	1.52–10.04	.005	2.05	.62–6.86	n.s

*CI* confidence interval, *CD* Clavien/Dindo classifications of complications, *ICU* intensive care unit, *HRFS* Hospital Frailty Risk Score, *mFI* modified Frailty Index, *OR* odds ratio, *prolonged LOS* median length of stay + 1 standard deviation, *n.s.* not significant

**Fig. 1:**
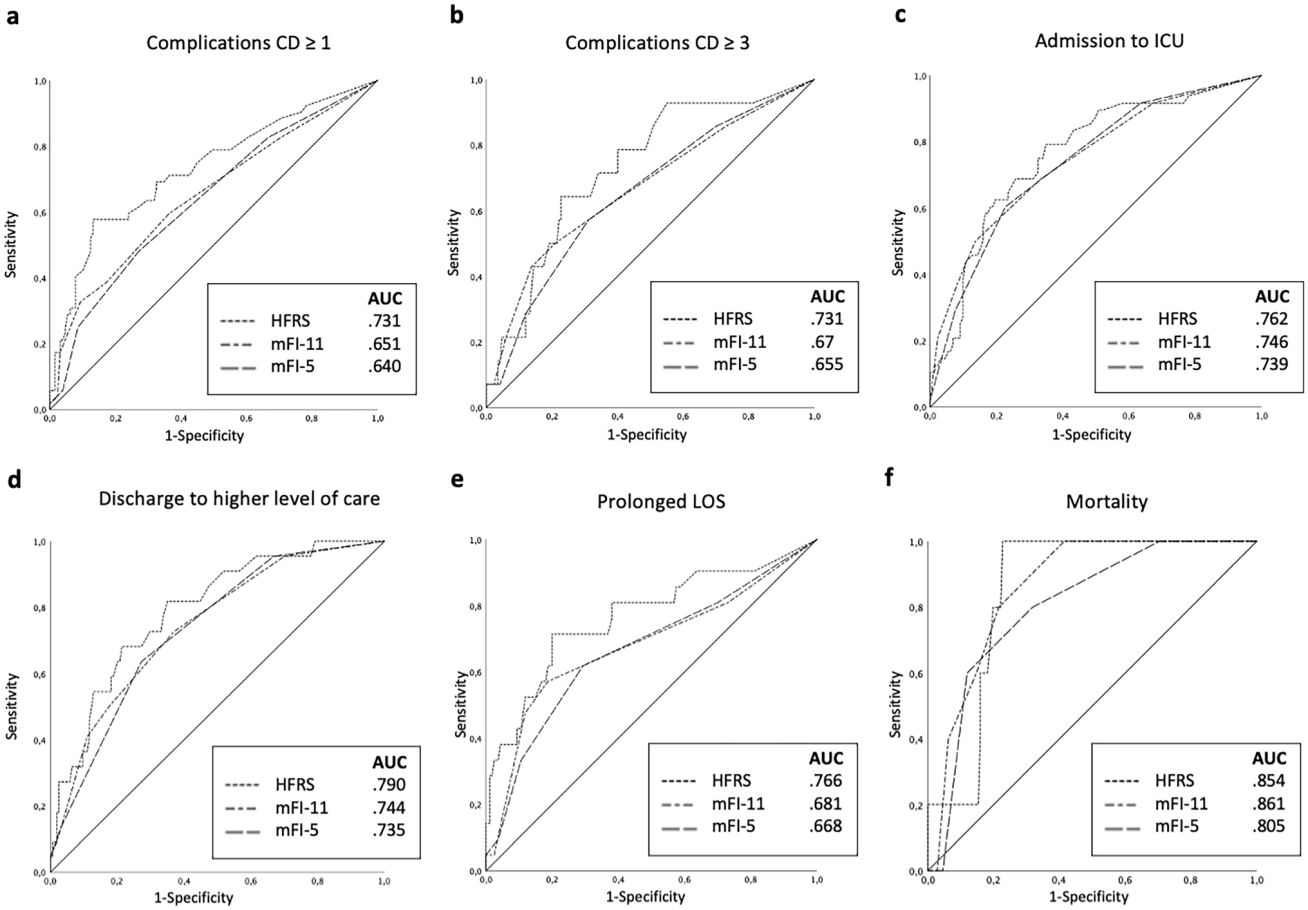
Receiver operator curves (ROC) of Hospital Frailty Risk Score (HFRS) and modified Frailty Index (mFI) 5 and 11 for complications according to Clavien/Dindo classification (CD) ≥ I; **b** CD ≥ III; **c** admission to intensive care unit (ICU); **d** patient’s discharge to a facility with a higher level of care than used before admission; **e** prolonged length of stay (LOS) = mean LOS + 1 standard deviation; **f** mortality

For the abovementioned assessments and outcome parameters, ROC analyses were performed in which changes in the LOS were analyzed dichotomously (prolonged LOS vs. non-prolonged LOS). The median LOS + 1 standard deviation (SD) was defined as a prolonged LOS (Fig. 1). The ROC analysis showed that the sensitivity and specificity of the three assessments did not differ in a relevant manner, and the same applies to the *area under the curve* (AUC) values as a measure of test quality.

## Discussion

It has been demonstrated that frailty is a critical factor in outcomes of medical treatments. Frailty can be objectively evaluated with assessments, of which multiple types with different focuses are available. In surgery, the evaluation of frailty is primarily used for preoperative optimization of frail patients. Usually, preoperative optimization is infeasible in emergency surgery, but peri- and postoperative therapy can be optimized. Of the more than 70 validated frailty assessments, approximately 30 are available for surgical patients but very few them are feasible in emergency situations [21]. Even the *Emergency General Surgery Specific Frailty Index* (EGSFI) requires the active cooperation of the patient, which is not always possible in patients with severe acute appendicitis [29].

Thus, two well-established assessments were selected for this study: (1) the mFI and (2) the HFRS. Both can be conducted based on information and findings routinely available in the emergency room. It was shown that assessments based on electronic patient records recognize and determine frailty just as well as bedside assessments [30].

The choice of assessments was to some extent arbitrary, but the results of the study confirm that useful choices were made. It was possible to prove the functionality of the two assessments and compare them to a certain extent, both of which could be carried out with limited effort post hoc by non-geriatric specialized physicians. Furthermore, we demonstrated that the shorter assessment, the mFI, delivers high-quality and accurate results and is clearly correlated with the most important outcome variables. This is interesting because compared to the HFRS, the mFI delivers significantly better test quality (high accuracy of diagnosis-transfer 96.7% vs. 17.1%, *p *< 0.0001) in less time (mean 21.6 s range 0–45 s vs. 80.3 s, range 0–220 s; *p *< 0.0001). It is known that the mFI-11 and mFI-5 are equally effective in predicting frailty, although the mFI-5 is one of the easiest and fastest frailty assessments available [25].

Studies on frailty in the context of emergency surgery can mainly be categorized into two groups. First, several studies analyze data from databases, e.g., the *American College of Surgeons National Surgical Quality Improvement Project* (NISQIP). Second, there are observational clinical studies [14, 15, 18, 31–33]. The latter included patient numbers comparable to our study, while the database analyses naturally used the data of tens of thousands of patients. As previously mentioned, there are no studies with a distinct focus on appendicitis. In contrast to database queries, our study allows the assignment of the ascertained assessment to an individual clinical course. We could record additional parameters and facilitate a follow-up of 90 days, both of which are clear advantages over a database query. Moreover, a database comparable to the NISQIP is not available for Germany.

Our study demonstrates the unambiguous association between frailty and negative outcomes in elderly patients after appendectomy.

In patients with a score indicating frailty (HRFS ≥ 5 or mFI-11 ≥ 3), complications of any severity were more than twice as frequent compared with patients without signs of frailty (48.8% vs. 22.9%, *p *= 0.003), and the OR was 5.9 for a HFRS ≥ 5 (95% CI 2.7–12.8, *p *< 0.0001) and 2.9 for an mFI-11 ≥ 3 (95% CI 1.3–6.3, *p *= 0.009). For serious complications (CD ≥ III), we observed a more than threefold increase in the complication rate (17.1% vs. 5%, *p *= 0.018) and ORs of 4.9 (HFRS 95% CI 1.5–16.7, *p *= 0.011) or 3.6 (mFI-11 95% CI 1.1–11.5, *p *= 0.029). The length of inpatient stay was almost doubled in patients in whom the HFRS or mFI indicated frailty (no frailty: mean 6.6 days; HFRS ≥ 5 and mFI-11 ≥ 3 mean 12.7 days *p *< 0.005), and the OR for a prolonged LOS was 4.4 (HFRS ≥ 5; 95% CI 1.4–14.2, *p *= 0.014) resp. 6.0 (mFI-11 ≥ 3; 95% CI 1.5—24.7, *p *= 0.013). These observations are well in line with data published by other authors on frailty in emergency general surgery [14, 15, 18].

Elderly patients with elevated frailty scores were admitted significantly more often to the ICU, and this observation aligns with other studies focused on emergency surgery [18]. In our cohort, patients with an mFI ≥ 3 were 5.5 times more often admitted to the ICU (mFI-11: OR 7.4, 95% CI 3.1–17.6, *p *< 0.0001; mFI-5: OR 6.2, 95% CI 2.7–14.1, *p *< 0.0001; HFRS ≥ 5: OR 5.2, 95% CI 2.3–11.5, *p *< 0.0001) than those without evidence of frailty in the assessments (Table 2). This observation is quite interesting given that appendicitis only very rarely leads to ICU-therapy in the general population.

Our ROC analyses proved the sensitivity and specificity of the assessments for the most important outcome parameters. We were able to show that the observed relationships between frailty and negative outcome can be observed not only in dichotomous utilization of the assessments with the respective cutoffs but also across the entire range of points in the tests. The AUC, a measure of test quality, was very similar between the assessments, which is very interesting in light of the considerably higher effort required to conduct the HFRS compared to the mFI-5.

We demonstrated that frailty after appendectomy is also correlated with an increased rate of discharge to an environment with a higher care level compared to before admission (HRFS ≥ 5: OR 4.5, 95% CI 1.5–13.3, *p *= 0.006; mFI-11 ≥ 3: OR 4.5, 95% CI 1.5–13.2, *p *= 0.007; mFI-5 ≥ 2 OR 4.6, 95% CI 1.6–13.3, *p *= 0.006). This finding fits well with data published by Murphy et al. who demonstrated a reduced rate of home discharges for patients with intermediate and high frailty scores [15]. We deliberately did not choose the criteria “discharge home” or “institutional” vs. “non-institutional discharge”, as some of the patients referred for appendectomy were already residing in care facilities or retirement homes. This result demonstrates an immediate and productive application of the assessments. The modalities of a future discharge of patients with appendicitis who show frailty should be discussed and planned early in the inpatient stay such as by involving the social service.

We could observe an increase readmission rates, solely for surgical problems and only in patients with an mFI-5 ≥ 2. Rothenberg et al. showed that frailty is a risk factor for readmission but they did not study emergency surgery patients. Interestingly, frailty leads to a doubling of the readmission rate in his study, which is very similar to our observation in the subgroup with an mFI-5 ≥ 2 [34].

One objective of conducting frailty assessment in surgery is to positively influence the patient´s outcome by improving her or his preoperative condition [35]. This is hardly feasible in the context of an urgent appendectomy, and “prehabilitation” is not possible in these patients. However, even in this context, there still exist some options. For example, it is known that frailty increases the risk of postoperative delirium, which in turn is associated with consequences such as extended stay in intensive care. This risk can be countered by appropriate anesthesia (e.g., avoiding benzodiazepines, controlled depth of anesthesia). Malnutrition is another risk factor correlated with frailty and poor surgical outcomes. Frail patients can be treated early with a protein-rich, supportive diet and nutrition counseling [36].

Moreover, as we demonstrated, patients who show signs of frailty are subject to increased complications. This can be countered with greater awareness and additional examinations, such as laboratory controls or sonographies, in the postoperative course. Physiotherapy, intensified remobilization, or respiratory therapy are further options that may reduce the risk of complications in frail patients.

Important information is also provided for resource planning, such as bed occupancy or intensive care capacity. Ultimately, a demonstrated connection between frailty and increased mortality has an important implication. For example, if an older patient with frailty is informed about a necessary operation for acute appendicitis, then perioperative mortality, which reaches up to 17% (mFI-11 ≥ 3) in our data, can be emphasized differently compared to a patient without frailty. If simple tests such as the mFI can quickly and accurately detect frailty in a patient, then this can also be an opportunity for a detailed geriatric assessment which can help identify specific risk factors that can then be addressed with targeted treatment [37].

To our knowledge, this study is the first to focus on frailty in elderly patients undergoing emergency appendectomy, but a number of papers have been published that address frailty as a risk factor after emergency general surgery. This supports the stability of our observations as the results are almost entirely coherent to previously published data.

Frailty as a multifactorial clinical syndrome is not caused solely by the summation of disabilities and comorbidities. Furthermore, while there is no defined minimum age for frailty, it is well established that frailty is a syndrome of the elderly [38]. The age threshold of 65 years designating elderly patients is also widely accepted [39, 40]. The mFI has been validated for people ages 65 years old and above. While the HFRS was developed using data from patients over 75 years old, it has been successfully used in patients aged 65 years and above [41]. Our analysis shows that HFRS tends to predict worse outcomes for both over 65- and over 75-year-old patients with frailty. In this regard, our work is highlighted by the fact that we only examined frailty in patients over the age of 65 at the time of the operation. Only a small fraction of study groups investigating frailty in surgical patients undergoes this restriction [31, 42]. If other authors examine frailty across the entire age spectrum, including young people, and analyze age as a covariate, then we consider this questionable.

As mentioned at the beginning, the two most widespread concepts frame frailty as the result of the accumulation of deficits or the reduction of capacities and resources. While the underlying biological processes are largely not understood, there are established associations between frailty, immune system alterations, and inflammation. Some studies use the term “inflamm-aging”. We see increased white blood cells, CRP, TNF-*α*, and IL-6 in people with frailty, but it is still debated whether these are the cause or consequence of frailty [43, 44]. In the context of our study, an analysis of these parameters does not seem meaningful since acute inflammation, such as that in acute appendicitis, necessarily leads to an increase in inflammatory biomarkers. Values independent of acute inflammation are not available for our cohort.

### Our study is not without limitations

First, our study is a retrospective analysis. However, since all the requested parameters were entered into the electronic patient records immediately during the treatment process and could not be modified, the risk of bias is minimal. It can be assumed that the results presented will be confirmed or even clearer if the assessments are made in real time while a patient is being admitted.

All assessments that use encoded disease data are prone to quality deficits in coding. However, since these coded data are crucial for revenues in the German health system, all employees in surgical clinics are very well trained in coding. Furthermore, at all participating centers, specialized accounting employees verify coding at dismission. Thus, it can be assumed that coding quality is very high, which minimizes potential bias.

The tests used focused on recording deficits and diseases. This leads to an overestimation of multimorbidity while motor, cognitive, social, and psychological factors are underestimated. As already mentioned, the latter factors can hardly be validly recorded in acutely ill patients and within the constraints of the emergency room.

The number of patients included is small compared to registry-based studies but is within the scope of the examined patients in comparable clinical analyses and the proportion of patients over 65 years is even slightly higher compared to that of epidemiological studies [2]. The data were collected from three independent hospitals and are very coherent with regard to all parameters. The statistical analyses were unambiguous in their statements. Only the low total number of deaths in the study population precluded the feasibility of a regression analysis of this parameter that would result in meaningful results.

The fact that old age is correlated with poor prognosis and outcomes in acute appendicitis is well documented in the literature and is hardly controversial. However, we were able to show for the first time in a clinical study that it is more so frailty, rather than age which causes poor outcomes.

## Conclusion

Frailty is an important risk factor for elderly patients with acute appendicitis. While this disease is associated with low morbidity and very low mortality in younger patients, in older, frail patients, we observe an outcome that is significantly worse than would be expected based on age or comorbidities alone. In this cohort, frailty can be reliably recorded using simple assessments based on data routinely collected by each surgeon during an emergency admission. Identifying vulnerable patients is fundamental for determining targeted countermeasures and for optimizing resource planning. The latter is crucial, as the importance of understanding and treating appendicitis in the elderly population is becoming increasingly evident.

## Data Availability

Not applicable.
